# A Telemonitoring Tool based on Serious Games Addressing Money Management Skills for People with Intellectual Disability 

**DOI:** 10.3390/ijerph110302361

**Published:** 2014-02-25

**Authors:** Asier Lopez-Basterretxea, Amaia Mendez-Zorrilla, Begonya Garcia-Zapirain

**Affiliations:** DeustoTech-Life Research Unit, DeustoTech Institute of Technology, University of Deusto, Avda. Universidades, 24. 48007, Bilbao, Spain; E-Mails: asilop_92@hotmail.com (A.L.-B.); mbgarciazapi@deusto.es (B.G.-Z.)

**Keywords:** serious games, multitouch devices, intellectual disability, telemonitoring

## Abstract

This article presents a telemonitoring tool based on computer games, aimed at money management skill improvement for people with Intellectual Disabilities (ID). The presented tool is divided into two parts: on one hand, some training activities related to payments and currency discrimination based on Serious Games are proposed to the user using a multitouch device. On the other hand, the psychologists and specialist who work with them, can access to the Serious Games results using an online application in order to evaluate their evolution. The results are measured according to the number of errors they have during the proposed activities, the time they need to complete them and the score. The article show the results of an experiment made with a clinical sample of 12 users with ID between 12 and 15 years, taking into account that all of them are capable of correct oral communication and they do not have severe physical coordination problems. Only two users completed all the games without errors. Males obtained a mean of 28.25 errors, whereas females obtained a mean of 17.75. The results show significant difference between the selection of games 1, 2 or 3, because all of them prefer the game 1 related with “Payments” probably because it permits more interaction using the multitouch device. The authors also made a qualitative evaluation and the results have been very promising and satisfactory.

## 1. Introduction

In Spain, approximately 3.78 million people have some kind of disability, either intellectual, mental or physical. This accounts for 8.9% of the population. The prevalence in the Basque Autonomous Community is 169,400 people with some kind of disability or 8.4% of the population [1]. Some 11.5% of working-age adults with certified disabilities, have intellectual disabilities (ID) in the Basque Association of Representatives of People with Disabilities [2] and nationally this figure is 13.6 % [3].

Intellectual disability is defined as “a disability characterized by significant limitations both in intellectual functioning and in adaptive behaviour as expressed in conceptual, social, and practical adaptive skills” [4]. Therefore, the limitations related to ID, in addition to intellectual functioning well below average, focus on the following areas: communication, self-care, home living, social skills, community involvement, autonomy, health and safety, functional academic skills, leisure and work [5]. In this regard, their main features are: impulsive behaviour, deficiency in discernment, short attention span and slow learning [6].

In addition, chronic health problems and comorbidity with other health problems are common. These problems include psychiatric abnormalities (such as mood disorders, anxiety disorders, behavioral problems [7] and psychotic symptoms when faced with stressful demands [8], chronic diseases (including epilepsy, diabetes, chronic constipation, gastrointestinal problems, thyroid disease, allergies and cardiovascular problems) and obesity [9]. In this sense, this group shows great heterogeneity both in symptoms and severity. In this variety, Down’s Syndrome (DS), which is a genetic disorder, is one of the most common categories of ID [10]. Furthermore, there is no cure for ID [5].

For these reasons, it is necessary to create resources for this collective, to promote independent autonomous and health living [11]. In this case, the authors have had previous experience working with people with DS and our interest centred on improving the capabilities of children with this disability. 

This article has a double aim. On the one hand, to provide people with ID (especially DS) with a set of Serious Game, which allows them to work on assimilating basic skills of daily life, money management and coin discrimination. On the other hand, to develop a telemonitoring tool which permits professionals to follow the results of people with ID they are in charge of.

In addition to the main aim, other secondary objectives are defined:

- Work on reducing the digital divide among people with disabilities.
- Design technological tools that deal with key habits for independent life
- Evaluate the results obtained and measure the satisfaction of people with ID concerning initiatives of this type.

Money management and healthy habits are the most discussed topics, taking into account the group’s needs, their difficulties involved in performing them and their need for an autonomous and independent life. 

To this end, some mini games/activities have been proposed with different objectives and difficulties. All of them are developed within the same application for the multitouch device (iPad), which, in turn, registers all the processes.

This article is divided into the following sections: Section 2 reviews the literature related to ID and Serious Games. Section 3 sets the context for the methods used, the participants and the design of the study. Section 4 explains the technical results and the results of the tests with participants, and, finally, Section 5 collects the conclusions of the authors after having conducted the experiment, followed by their reflections.

## 2. Background

This section reviews the previous work available in the literature on interventions for ID and the use of Serious Games for Health in the field of ID.

### 2.1. Interventions for ID

As a result of the symptoms described in the previous section, people with ID often experience serious difficulties to perform daily activities independently

It is particularly relevant that people with ID should be as fully integrated as possible into the daily routines of people in society. The Down’s Syndrome Association in Bilbao, with which we have collaborated on this project, has a special “Independent Living” programme where they offer their users the possibility of living in rented flats shared with 3 or 4 other people with ID. Although there is the figure of a “support supervisor”, they need to be able to perform their own daily routines like shopping, personal finance, hygiene, *etc.* and, therefore, it is essential to provide them with previous training [12]. It is in this framework that the research project described in this paper has been proposed and tested [13].

In this regard, to cover the greatest number of limitations, the support and tools should come from multidisciplinary and multifactorial interventions, where the use of new technologies should also be present [5]. Nowadays, the main specific technological resources focus on prenatal detection and diagnosis, screening tests and the study of genetic characteristics. However, given the characteristics of the group, very few ad-hoc experiences, projects and methodologies have been developed for them, in most cases all those existing for other groups have been applied without adapting them [14].

Therefore, taking into consideration the previously mentioned features and related points, new techniques are needed to enhance the effectiveness of the activities they must perform. Specifically, it is in those aspects related to learning and health that ad-hoc developments applying new technologies can be useful. In this approach, Serious Games and telemonitoring should be combined. What is more, according to [15], the new technologies offer the possibility of education and learning through interactive games in a stimulating atmosphere. 

### 2.2. Serious Games and ID

Serious Games are a very useful problem-solving tool, since, in addition to offering entertainment to users, they are also educational. This link between leisure and education makes the experience more positive and enriching than other learning methods [16]. So far, the projects designed for people with DS or ID, in general, have focused on the acquisition and improvement of language and communication skills, using serious games as a tool [17].

In these experiences, people with disabilities were hardly taken into account in their design and conception, nor were they given feedback about the work done or progress made. The concept of remote monitoring or telemonitoring allows monitoring the activities performed by the user, collecting objective data, measuring progress, checking the correct functioning of an application [18], and even sending messages to the user to provide him/her with feedback and information.

The use of these techniques are becoming more frequent, since they offer many interesting data and statistics that both instructors and expert psychologists can analyse in a more objective way [19].

In the literature, there are many projects in which telemonitoring is used in general. They are made for many different purposes, mainly focused on health monitoring in chronic patients: diabetes, hypertension, COPD, *etc*. [20,21,22]. Conversely, there are not many references to telemonitoring applications oriented towards people with ID, and no studies of this kind were found if we add the Serious Games variable on multitouch devices. 

Table 1 shows some examples of applications found in the literature and market. To complete the table the following points have been taken into account:

- The technology used : the software on which the application has been developed.
- Whether the system has telemonitoring services, or not.
- The target audience at which it is aimed.

Now we will examine the most important issues of the Table 1. Regarding technology, it is evident that the use of multi-touch devices is preferable to the use of PCs (mainly in the most recent developments). It is in the examples from 2010 and 2011 that some were done for PCs.

The authors were surprised by the fact that very few applications provide telemonitoring services, in order to monitor the results of each user while playing. In the literature, the majority of applications that use telemonitoring are related to the treatment of chronic patients with or without disabilities.With respect to user profiles, we should point out that the applications are aimed, in general, at the specific characteristics of a group and therefore, the games/applications do not require a smart engine that covers different user profiles.

Two clear trends can be observed in the developed applications, one that is exclusively leisure-oriented, and another has a broader scope, where the goal goes beyond the game itself and focuses on training activities, both physical and mental.

**Table 1:** Application examples.

Títtle	Authors	Country	Year	Objective	Technology	Telemonitoring?	Oriented to?
Picaa [23]	GEDES from the University of Granada	Spain	2010–2011	Creation of learning activities	iPad, iPhone and iPodTouch	NO	ID
Lucas y el caso del cuadro robado [24]	Orange Foundation and Madrid Down Syndrome Foundation	Spain	2010	Leisure Game	PC	NO	ID
Las aventuras de Spoti [25]	Down Spain and Eroski Foundation	Spain	2011	Online interactive nutritional game	PC	NO	ID
Sudoku [26,27]	N/A	N/A	2012	Mental Exercise	IPad	NO	Elderly
Bowling [28]	Sunwoo, J.; Yuen, W.; Lutteroth, C.; Wünsche, B	N/A	2010	Physical Exercise. Easy bowling game in which the user has to imitate the movement of throwing the ball.	IPhone	NO	Elderly
Penguin toss [28]	Chris Hilgert	N/A	2010	Physical Exercise. In this game, the user has to move the smartphone in order throw the penguins through the rings.	IPhone	NO	Elderly
Proloquo2Go [29]	AssistiveWare	N/A	2013	Symbol support—Voices	iPhone / iPad	NO	People in general
Autism Colors [30]	Bob Bradley	N/A	2012	Learn basic colors	iPhone, iPad and Android	NO	Autism children
TapToTalk [31]	Assistyx LLC	N/A	2013	Help people who are non-verbal clients or with limited speech	Tablets and PC	NO	Children
Sutil+ [32]	Barro Chris Hilgert, S.; Presedo, J.; Castro, D.; Fernandez-Delgado, M.; Fraga, S.; Lama, M.; Vila, J.	Spain	2002	Monitoring of patients in coronary care units	N/A	YES	Critical care patients
Real Time Patient Tele-monitoringSystem Using LabVIEW [33]	Mr. Bhavin Mehta, Ms.Divya Rengarajan, Mr. Ankit Prasad	India	2012	Provide doctors patients vital parameters	LabVIEW and online	YES	Cronic patients

## 3. Material and Methods

In this section, the resources used (both human and technical), the design of the experiment and other key points are described.

### 3.1. Participants

A clinical sample of 12 people with ID from the Down’s Syndrome Foundation in the Basque Country (FSDPV) took part in this study, all of whom are of school age and whose informed consent was signed by their parents. The inclusion criteria for these participants were as follows: (a) be in early integration or independent life preparation programs; (b) be capable of correct oral communication; (c) be 12–15 years of age; (d) form part of a support/school group.

Exclusion criteria included: (a) lack of capacity to respond to the questionnaires proposed for the assessment due to severe communication deficiency; (b) severe physical coordination problems which hinder use of the multitouch device; (c) verbal or written refusal.

A group of intellectually disabled persons were chosen for the trials. The sample was constituted by a group of adolescent girls (*n* = 4) and boys (*n* = 8), and they showed varying degrees of ID (not only DS). Most of the participants are members of the Learning for Independent Living program of Down’s Syndrome Foundation in the Basque Country (FSDPV), led by Laura Fernández. Various supporting instructors as well as the system developers also participated, both during the tests with the children and providing information concerning the application content.

### 3.2. Informed Consent—Institutional Review Board (IRB)

The Research Ethics Committee standard was followed when carrying out this study. Parents of the potential participants were duly informed about the research aims [34,35]. Additionally, signed informed consent of the children’s families was required for them to take part.

### 3.3. Human Resources

This study was possible thanks to the cooperation of a multidisciplinary group formed by technicians, psychologists and people with ID, who participated in all the stages, from design to validation of the telemonitoring application. It should be mentioned that this project has involved the entire value chain in the research from the start:

- Prescribers: the supervisors and therapists from the association of people with ID.
- ICT experts: experts in the design and development of the tool.
- End users: people with ID in the association. They all have shared a key role in the specification of the tool [36,37].

Prescribers mainly at the functionality definition phase; end users in the validation of the proposed interface that should be friendly and approachable; ICT researchers in the translation of the technical language of functional specifications.

The development phase was led by ICT researchers, but pre-assessments were required from prescribers. Finally, all the above mentioned elements of the value chain of this project participated in the final validation phase of the tool.

### 3.4. Accessibility and Usability Criteria

Close attention was paid to the application interface, which was designed especially for children with ID [38]. These are some of the most relevant points:

- Increased font size.
- Voice accompaniment in texts and messages, guiding the user.
- Use of realistic elements to simulate a real case.
- Clear and easy interfaces.
- No time limits. Due to differentuser profileswithin theID, there are no time limits to perform the activities. In addition, the aim is to encourage the useofthe application, not to prevent or hinder it.
- Short conclusions after each Game to encourage users and facilitate the learning process by repeating concepts.
- Lack of animations and sound effects to keep the attention of the user.

**Figure 1:**
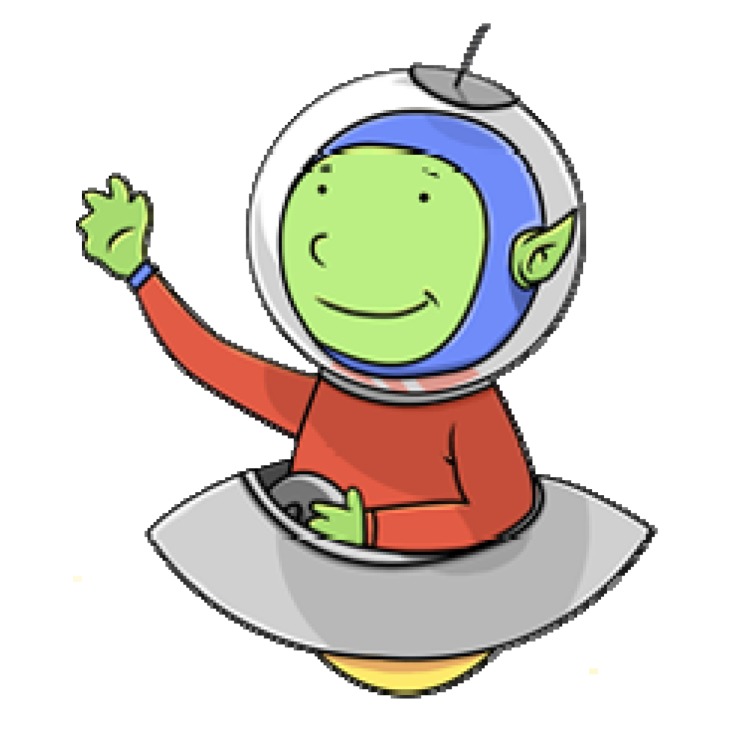
Main character of the System.

Furthermore, the design of main character for the system was performed by involving the entire value chain described in Section 3.3. The aim was to ensure that the user of the game saw the character as a close, friendly and funny friend, as shown in Figure 1. 

### 3.5. Experiment Description

During the design of the project, initial meetings were held with the heads of various programmes. These professionals work directly with members of the association (FSDPV). These meetings enabled us to choose the hardware device, to correct some ideas and concepts, and also to adapt the development of the application to the needs of the final users more easily. All the experiments have a similar structure, and complete tests were conducted altogether, alternating them with short demos. The variables studied were: time, number of attempts and errors. All the sessions, both for the design and validation of the system have been carried out in the FSDPV facilities.

### 3.6. Methods

This section describes all the methods software and hardware used to develop and assess the tool.

#### 3.6.1. Technological Methods

The technologies used in this study are detailed below. Firstly, The system is composed of two databases: one that stores data locally on the iPad, developed in SQLite and, on the other hand, the database to which all data are sent over the Internet and is available on the platform to which psychologists and specialists access, developed in MySQL. Secondly, a programming language known as Hypertext Markup Language (HTML5) was used to develop the activities included in the online telemonitoring system. The training activities based on serious games were developed for the iPad platform, property of Apple, based on IOS. Thus, the code programming is carried out through the Apple’s own environment: Xcode.

#### 3.6.2. Hardware Device

The multitouch device (iPad) was chosen due to its mobility, multitouch screen, multimedia capacity and interaction through movement, which are interesting features to take advantage of with the application.

The chosen device is user-friendly, regardless of the user’s intellectual capacity, or reduced motor skills, as it is a 100% digital device. Furthermore, it is considered that a proper platform to provide stimulating training, as its use is intuitive and interactive [39].

#### 3.6.3. Evaluation

The results were evaluated twice. On the one hand, an adapted questionnaire (see Table 2) was created, along with the people working in the association, to measure the satisfaction of people with ID regarding the proposed system. This was a qualitative evaluation, and the feedback obtained will be described in the results section. The questions asked to the users after the tests are shown in Table 2:

**Table 2:** Satisfaction survey.

Item ID	Question
A	Did you like Kimi?
B	Did you have fun?
C	Will you play again?
D	Did you find the game complicated?
E	Did you enjoy the music?
F	Did you know/ have you ever played with the iPad?
G	Do you use a mobile phone?

As shown in Table 2, all the questions were asked orally in a very clear and simple way to facilitate the understanding, so all users, independently of their reading and writing skills could understand them. The G item was asked to know the degree of access to the technology proposed by the sample of participating users. All the items were evaluated with YES/NO answers, because all the users were not able to assess the level of difficulty from 1 to 5. On the other hand, an objective assessment based on parameters included in system was made: time spent in performing the proposed game/activity, number of attempts or level played.

## 4. Results

In this section the technical results of the development of the application are explained in detail, as well as the objective results about users’ performance on the serious games of the application during the trials.

### 4.1. Tool Design

The complete telemonitoring tool is divided into two main parts: online application for specialists and instructors who work with the final users, and serious games part, based on a multitouch device development.

#### 4.1.1. High-Level System Diagram

The proposed application has a three-layered structure:

- Database (internal and online). The various multimedia elements making up the application are situated in this layer. They display and capture the user information in order to deliver it to the business layer.
- Logical layer. The operations to perform for the application’s functioning are executed in this layer. They are collected and the information is sent to be displayed in the presentation layer and the information from the database is stored or obtained.
- User interface. This layer is composed by the database manager, which will back up the business layer in the storing and data-obtaining processes.

**Figure 2:**
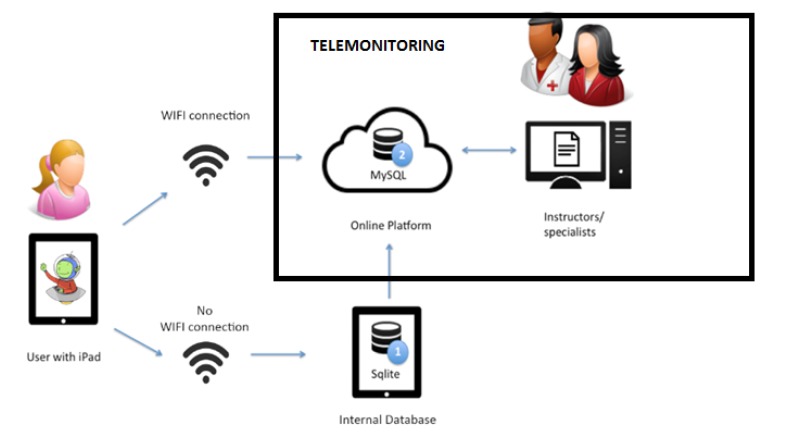
System Architecture.

The following structure is used for content synchronisation and communication and permits instructor and specialist telemonitoring (Figure 2). As it can be seen in Figure 2, two database architectures have been used: Sqlite and MySQL. The former, Sqlite (1), is implemented in the multitouch device (iPad), and ensures that data are stored, even without an internet connection. The latter, MySQL (2), which is employed on the Online Platform and has with a similar design, offers an online service that, together with the previous database, permits having all the information synchronized (offline and online).

Regarding the operation in general, as the user plays the game with the multitouch device (previously connected to a WIFI network), data are sent to a server with a MySQL database. If at that time the WIFI connection is not possible, the application simultaneously saves all data in the internal database and synchronises it later, when Internet connection is possible.

#### 4.1.2. Online Application

Specialists/psychologists have access to the collected data by using the online application for telemonitoring, which can be personalised, allowing them to visualise the results of users who they are in charge of. This application collects data about the following variables: attempts taken, the time needed to complete each game and the mistakes made by the users. The telemonitoring tool makes it possible to manage all the data received and collected from the users, and also to extract statistics, and make reports. 

By analysing all the information provided by the telemonitoring tool, the specialist can decide in a more knowledgeable manner which intervention to follow for each person. The online application defines different user access profiles:

- Specialists/psychologists
  These are the persons in charge of one or several users with ID. They will have access to the objective data the application receives and can monitor the development/learning of each user. Evolution is shown by weekly intervals and the data may be shown as numbers and graphs.
- Administrator
  The administrator is the person(s) in charge of making sure the tool runs smoothly, signing users on or off (both specialists and adolescents with ID). The administrator can never visualize personal and sensitive information, thus safeguarding the privacy of all the users.

**Figure 3:**
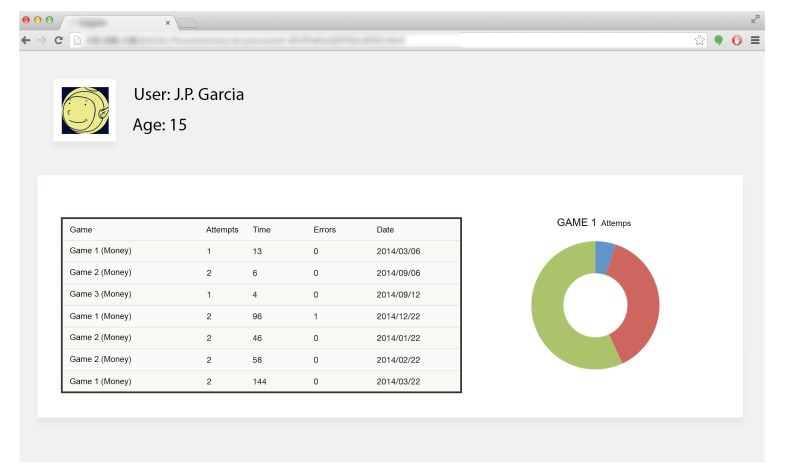
Online Platform screenshot.

In the following screen capture (see Figure 3), we can see a screenshot of the online platform. Specifically, in this example it is shown what obtained data a psychologist registered in the system about the user who is in charge of. As it can be seen, the data recorded on the table are sorted by date, allowing professionals and instructors to assess the results and draw conclusions graphically based on objective data, as previously shown. 

#### 4.1.3. Serious Games Part Design

In the mini-games the themes of money management and coin discrimination have been addressed. The importance of differentiating the coins has been highlighted, distinguishing between euros from cents and euro coins from foreign coins, in an attempt to increase users’ knowledge and their confidence when handling money in real life.

**Figure 4:**
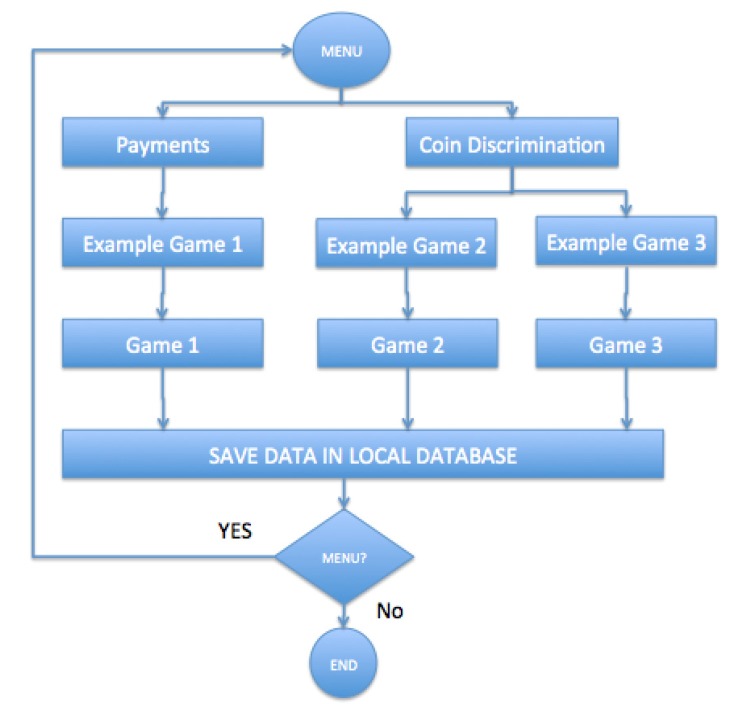
High Level Block Diagram.

In Figure 4, it can be observed that the Serious Game part has been differentiated into two blocks: payments and coin discrimination. 

In the “Payments” block, there are the games related to coin management when making a payment. In contrast, the “Coin discrimination” block includes activities to distinguish coins (colour, shape, value...). In both cases, when an activity ends, the objective data on the games are stored in the local database, waiting to be connected to the Internet so that they can be sent to the central system.

In addition, it is always possible to go back to the menu, to ensure that users do not get tired. Each game contains an example prior to each activity, with a guide in audio and text, explaining the objectives and how each game works.

**Figure 5:**
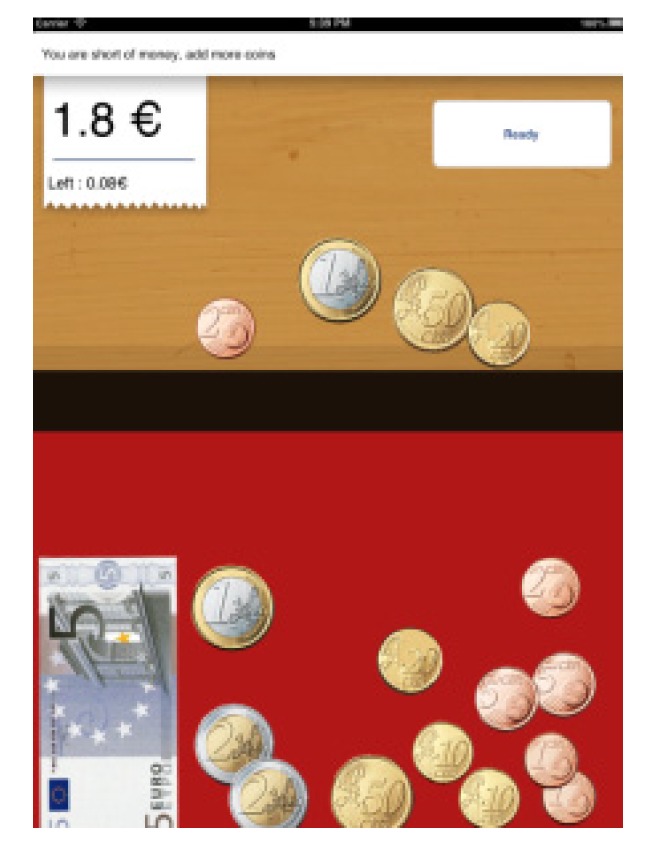
Game 1, block A.

**Figure 6:**
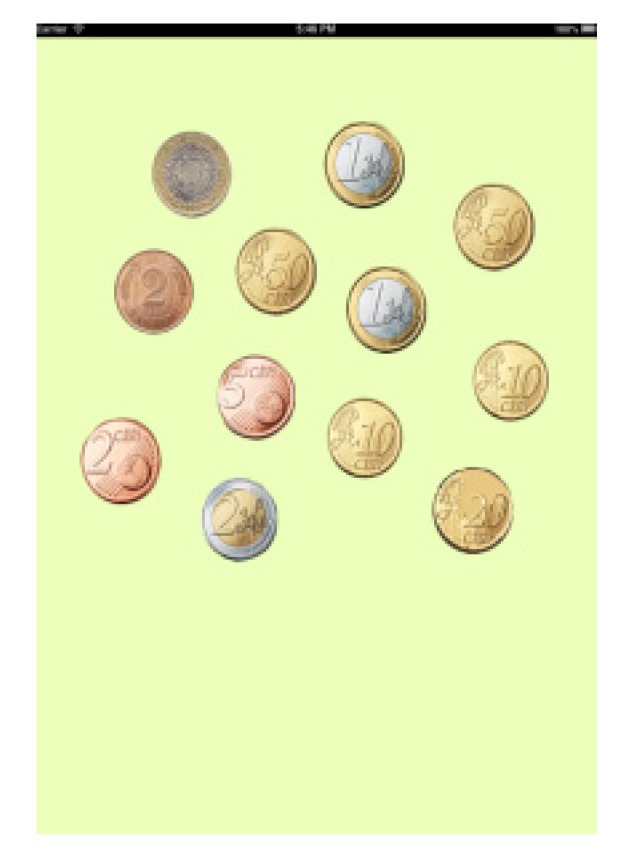
Game 1, block B.

**Figure 7:**
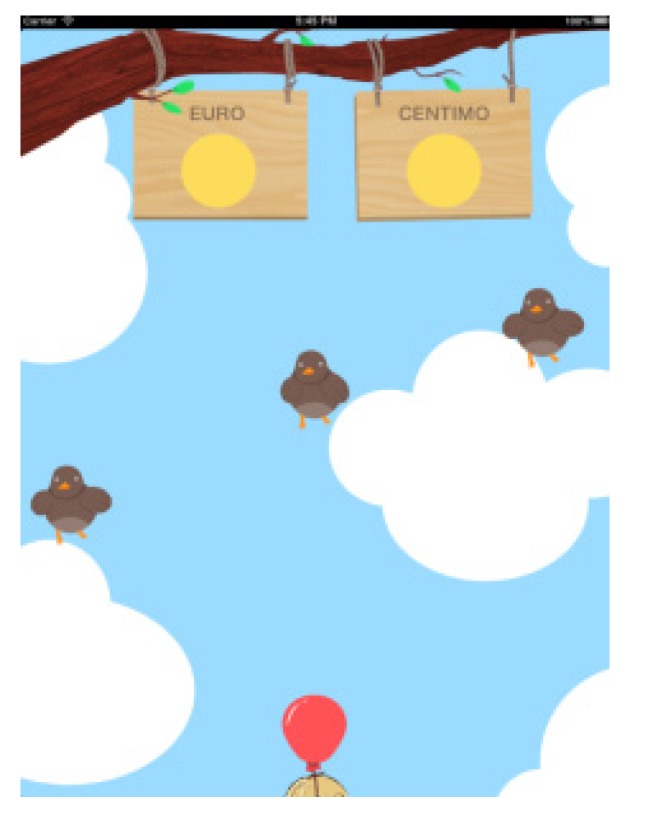
Game 2, block B.

Each game is summarized below:

- In the first game (Game 1, Payments), the aim is to reach a certain amount of money that appears randomly. The player uses two coins from the entire range (€), and a 5-euro note. The aim is to reach that amount without exceeding its value, avoiding one of the most common mistakes this group makes when paying. The game has three different levels, depending on whether the user receives help or not (see Figure 5):
  - Help indicating whether there is too little or too much money and how much.
  - Help indicating only if we have spent too much orfallen shortofthe amount shownwithout indicatinghow much.
  - No help at all.
  In Game 1 in all levels, the data on the number of attempts made are saved until the correct amount is selected.
- In the second game (Game 2, Coin Discrimination), the main aim is to distinguish between euros and other foreign currencies, which are randomly shuffled with each other. Users have to differentiate values, shapes and similar colours. In this game, the total amount of time spent in performing the activity was also calculated (see Figure 6).
- In Game 2, the number of timesthecoins thatwere not the sought oneare registered. In the third example (Game 3, Coin Discrimination), the aim is to distinguish between euros and cents, making use of the iPad’s gyroscope. Introducing this type of technologies entails increased user leisure as well as variety within the same thematic block (money) (see Figure 7). In Game 3, the mistakeson the number ofcoinsthat the user is offered to distinguish are saved.

In all cases, the time spent on the activity is taken into account, although *a priori*, there is no set time to complete each of them.

### 4.2. Objective Results

The results presented in this section are the results of the users’ scores stored in the database. These are shown in Table 3, as well as basic socio-demographic data. 

**Table 3:** Sociodemographic data of the sample and stored scores of the trials (12 users).

Parameter	Frecuency (*n* [%])	Average (DT)	Min/Max
Gender			
Female	4 [33.3]		
Male	8 [66.7]		
Disorder			
Down’s Syndrome	9 [75]		
Other ID	3 [25]		
Game (User Results)	*	**	**
Game 1	11 [43.83]	2.42 (1.31)	0/5
Game 2	6 [25]	1.58 (2.54)	0/8
Game 3	7 [29.17]	0.67 (0,65)	0/2
Total errors (score)		24.75 (29.25)	0/97
Total errors for female		17.75 (22.87)	0/48
Total errors for male		28.25 (32.83)	1/97

* *n* of players who played each game regarding the whole group, the percentages do not relate games with each other. ** Data about the no. of attempts in which each user has played each game.

As it can be seen in the Table 3, regarding gender, just over 33 % of the 12 adolescents who participated in the trials were female and two thirds male. In addition 75% of them had DS and the rest of them had other forms of intellectual disorders. So the sample was clinical.

**Figure 8:**
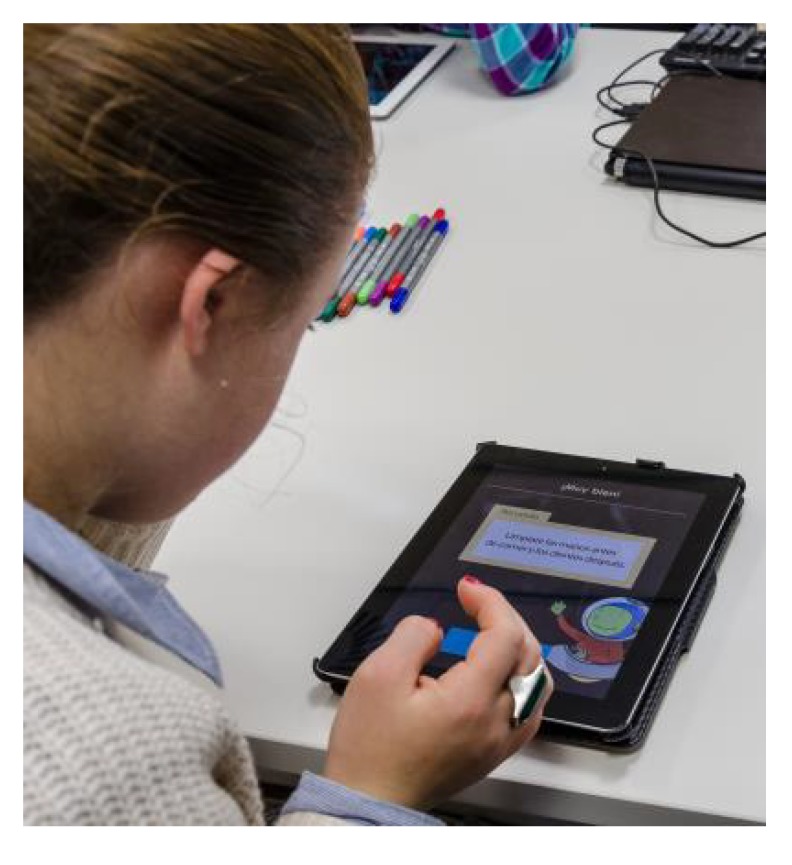
A child with ID tries the application.

The Game section of Table 3 shows users’ results obtained with the applications, where it is observed that the first game is the one that most users played (approximately eleven out of twelve) and the one that they played more frequently (mean 2.42 attempts). Games 2 and 3 showed to be less popular, as only 6 and 7 times, they have chosen to play it, respectively. Moreover, although there was a case that played the second game 8 times, the mean of times that each game was played by each person was 1.58 and 0.67, respectively. However, there were some differences between females and males there were not statistically significant. Thus, it is important to note that the users decided freely which game to use, and how many times to try them, as shown in Figure 8.

Besides the data about the attempts in each game, in Table 3, the number of errors can be seen. Despite not shown completely in the table, only two users completed all the games without any errors, both of them were female. Males obtained a mean of 28.25 errors, whereas females obtained a mean of 17.75. This difference was not significant, either.

### 4.3. Subjective Results

After analysing the results of the questions in Table 2, it can be observed that the character obtained a great acceptance (100%) and that all the users had fun playing, and even they would play again. No significant differences were observed between boys and girls, or among users of different ages. 

As far as the difficulty is concerned, no remarkable difficulties were reported, but, the case of one of the users must be highlighted, who, apart from DS, suffers from pervasive developmental disorder (PDD), and explanations and additional support were needed in this case, provided by the application. 

Among the users participating in the test, it can be seen that some of them already knew, or they had already played with the multitouch device (iPad), as well as mobile phones. Nevertheless, it is not reflected in the results any type of advantage or better punctuation.

According to subjective results, all the users completed the test without difficulty, enjoying the format and the contents. It is important to note that the iPad never hindered and even encouraged the process. On the other hand, instructors and professionals in charge of the users expressed their satisfaction and even provided ideas for future updates.

## 5. Conclusions

Both professionals and relatives of children with ID, especially with DS, work and search for alternatives to improve their daily life and foster their independence. However, the literature shows that technologies are able to help them. In the proposed case, the aim was to design, develop and evaluate a technological tool based on Serious Games oriented towards people with ID and propose a new way for supporting them with one of their main weaknesses in their daily life: money management. At the same time, this tool should have worked as a telemonitoring device. This study shows that it is possible to develop this kind of technological solutions that work successfully in as a enjoyable training tool and having telemonitoring functions.

Furthermore, this study helps to answer the following questions: Can a successful leisure experience be achieved as an end in itself through use of an iPad? Can people with disabilities also enjoy their leisure time with the help of this device? Can we use a tablet as a telemonitoring tool? The results indicate that people suffering from DS and other ID respond in a very positive way to the developed application and also to the multitouch device used. This paper also shows that of the three games, Game 1 was the most successful one, as far as participation is concerned. In fact, this is particularly interesting as this game is the most complex and challenging one. 

We believe that this project is another step towards the normalization of the use of digital devices among people with ID and people, whether professionals or not, around them. In addition, the application addresses money management habits which are considered essential for independent and autonomous life. This is because having money management skills means opening doors for them to a more normalized life:

- Shopping, having a coffee, *etc.*
- Be employed in regular jobs in addition to special job centres.
- Finally, responding to the last aim, this means assessing the results on satisfaction of people with ID and people working with them concerning initiatives of this type, during this experience gratitude and appreciation have been observable, showing how important the community around people with ID consider this kind of initiatives.

As can be observed in Section 3, the following important points should be highlighted:

- In the subjective questionnaires, it can be observed that the users experienced pleasant sensations and enjoyed playing the games. The professional instructors also expressed positive views.
- The results of the database reflect that the complexity of the levels is appropriate, since a wide range of results were obtained.
- The design of the application structure is considered to be appropriate, since the users were able to understand and move throughout the different sections.
- The use of an iPad or a Tablet with similar features to send the data collected from the Serious Games to the telemonitoring tool is a good option.

Furthermore, taking into account that is not possible to recover from ID [5], all effort and interventions have to be oriented towards improving the quality of life of people of this collective as much as possible and promoting autonomous and normalized life [11]. This study contributes to developing resources to make this possible. Based on our results, we suggest that tools for intervention that focus on instilling skills training via technological devices and through leisure could be a step for a more independent life in this population, though more research is necessary. 

The main limitation of this study was the small size of the sample. There were 12 participants and all of them were recruited from Down’s Syndrome Association of Bilbao, most of them had DS and they were mainly male. This limits the generalizability of the findings. To solve such limitations, future studies should recruit a larger and more balanced sample for the trials. Nevertheless, the sample is considered sufficient for this study taking into account its clinical nature and the purpose of our piece of research.

Another important limitation is the cross-sectional and laboratory condition of the methodology, which does not provide us with information about the training and development of users of the application. Thus, not only is the social validity of the tool limited, but also its generalizability. Future research with longitudinal design and consisting in natural environment is therefore necessary. However, regarding methodology, it is to be noted that this study does take into account the personal point of view of individual using qualitative designs, which as Diener considers, is necessary [40].

After having conducted this project, the authors have defined some other additional lines of research:

- To develop new Serious Games to work on other abilities and skills.
- To assess the application orienting it to wider groups of users, such as people without disabilities, and compare the results of different collectives.
- Add a wider variety of coins and notes into the games.
- Design and develop activities oriented to a real scenario, such as shopping in the supermarket.

To summarize, while caution should therefore be taken in understanding and interpretation of data, this research is valuable as one of the few studies conducted about serious games with telemonitoring features that are oriented to people with ID.
